# Van Wyk-Grumbach syndrome and its clinical heterogeneity: A case report

**DOI:** 10.6026/9732063002001317

**Published:** 2024-10-31

**Authors:** Krisha Jain, Shreya Siera Honarius, Reema Reji, Ravi Sangoi, Saurabh Mutha, Mayur Wanjari

**Affiliations:** 1 Department of Internal Medicine, Government Medical College and General Hospital, Baramati, Pune, Maharashtra, India; 2 St. John's Medical College, Bangalore, India; 3 Department of Paediatrics, KM Cherian Institute of Medical Sciences, Kallissery, Chengannur, India; 4 Department of Research, Datta Meghe Institute of Higher Education & Research (DMIHER), Sawangi, Maharashtra, India

**Keywords:** Hypothyroidism, endocrine disease, precocious puberty, menarche

## Abstract

This case report presents a 6-year-old female with Van Wyk-Grumbach syndrome, highlighting its astounding clinical presentations and the challenges in its diagnosis and management. Although the disease is rare, Van Wyk-Grumbach Syndrome can have a devastating and serious impact in young boys and girls by affecting their hypothalamic-pituitary-gonadal axis. The enhancement in the medical knowledge has shown promising results in the complete treatment of the condition and superior diagnostic accuracy with suitable and necessary interventions. Treatment of Van Wyk-Grumbach Syndrome remains solely through the hormone replacement therapy of thyroid, which usually leads to complete relief of symptoms. This case highlights the unusual clinical presentation of a young female patient with Van Wyk-Grumbach Syndrome, challenging the existing diagnostic profiles and highlighting the need for personalized assessment for the patient. Since the diagnosis of this syndrome is based on the USG, MRI, X-Ray findings, enhancement in these imagining techniques will lead to increased accuracy and reduced delay in its diagnosis. This case aims to enhance the understanding of Van Wyk-Grumbach Syndrome, its medical heterogeneity and the need to advance its diagnostic and interventional prospects.

## Background:

Van Wyk-Grumbach Syndrome (VWGS) is generally characterised by clinical presentation of juvenile long-standing hypothyroidism, delayed bone age and precocious puberty which reverses to prepubertal state after receiving proper therapeutic interventions [1]. Although the condition isn't encountered frequently in clinical practice, the heterogeneity in its presentation may lead to inaccurate or delayed diagnosis and thusly may worsen its prognosis if not identified by a well-defined mechanism. The patient, if female, commonly presents with the chief complaints of vaginal bleeding and uncommonly with galactorrhoea or breast development. This early onset puberty like symptom is not accompanied with growth of pubic and axillary hair. Boys commonly present with macroorchidism without the known signs of virilization [2]. Long-standing hypothyroidism is the salient feature used to diagnose the syndrome along with delayed bone age and elevated levels of Thyroid Stimulating Hormone (TSH) [3, 4-5]. The nature of precocious puberty is isosexual and often incomplete in children suffering from VWGS [5]. Recent advancements in the imaging techniques including USG and MRI have lead to an increase in accuracy of diagnosing this condition. Other than the thyroid hormone replacement therapy, there is no other curative treatment for VWGS. We demonstrate a case of Van Wyk-Grumbach Syndrome in a 6-year-old girl who exhibited vaginal bleeding with no pubic or axillary hair and breast enlargement (galactorrhoea). In radiological imaging, she was found to have enlarged and left multicystic ovary along with diffusely enlarged pituitary gland.

## Case Presentation:

A 6-year-old female presented to the outpatient department (OPD) in our hospital with chief complaints of vaginal bleeding and progressive breast enlargement since past three months. The vaginal bleeding was not associated to puberty as the girl's pubic and axillary hair has not grown. She was second child of a third-degree consanguineous marriage, delivered vaginally at full term with a birth weight of 3600 grams. She has no past history of abdominal pain, weight loss, cold intolerance or constipation. There was also no history of long-term drug intake or radiation exposure. The patient had a normal Denver II in the Developmental screening test. In family history, the paternal grandmother has goitre and both the parents are short-statured. There is no family history of ovarian cysts, precocious vaginal bleeding or menarche and other malignancies or surgeries.

The patient has a short stature and has normal weight as per BMI. Her height was 96cm (percentile), weight was 20.4Kg (50th percentile). Her pulse rate was 66/min and blood pressure was 96/62 mmHg. She had a fairly pale, dry and scaly skin. Pallor was also appreciated in the patient during the general examination. According to the Tanner's staging of sexual maturation, she measured stage 4 for breast development and stage 2 for pubic hair growth. Her visual acuity and field were reported normal.

Starting with the initial investigations, the patient appeared in stable condition and had normal vital signs. Laboratory investigation consisted of CBC (Table 1) which suggested normocytic normochromic anaemia with haemoglobin level of 10.6 g/L (12.0-16.0). Patient's hormonal investigation revealed TSH 150 µIU/ml (0.35- 5.5), T3 45.5 pg/ml (60-181), T4 2.0 ng/ml (4.5-12.6), through radioimmunoassay technique, and electrochemiluminescence immunoassay revealed FSH 5.78 mIU/ml (0.3-2.0), LH < 0.07 mIU/ml (0.1-6.0) and prolactin 174.8 ng/ml (2.8-29.2). Radiological investigations consisted of a USG-abdomen and pelvis. The investigation (Figure 1 - see PDF) revealed that left ovary contains multiloculated cyst of size measuring 55 x 20 mm. An MRI imaging pituitary in the Sella Turcica displayed diffused enlargement of pituitary gland. An X-Ray scan of the bones of the wrist suggested that the bone age was delayed by 2 years. Microbiological tests conducted to screen for infectious diseases did not provide any significant findings.

After establishing the diagnosis of VWGS, the patient's therapeutic and management plan was initiated which focused on treating the hypothyroidism rather than a palliative treatment. This included Thyroid hormone replacement therapy with L-thyroxine daily dose of 50ug, given once a day at morning was prescribed. The patient's response and the compliance to the therapy were monitored closely. A significant and gradual improvement was seen in the patient through the course of her treatment. The frequency of vaginal bleeding was gradually regulated. The patient had significant clinical improvement which was assessed on follow-up where her weight was 23.2kg (75th percentile), height 101cm (percentile), normal thyroid functions with FT4 13.42 pmol/L (9-20) and TSH 1.088 ulU/ml (0.4-6) and the pelvic USG taken recently revealed complete regression of left ovarian cysts suggesting a recovery. During patient's follow-up, the thyroid functions normalized at 5 months.

## Discussion:

Hypothyroidism is well known to cause retarded physical and mental growth of a person. Long-standing hypothyroidism in prepubertal girls may lead to the development of multiple cysts in ovaries which may have active hormonal repercussions. In a prepubertal child, this may result in incomplete isosexual precocious development [6]. These manifestations are known to subside by normalization of the circulating thyroid hormones in the blood. This includes complete resolution in terms of halting precocious development and allowing body to delay the complete development of secondary sexual characters to the time of puberty [7].

Effects of hypothyroidism in male gonal functions and its impacts on the reproductive system are not widely known. Although some studies point out the role of hypothyroidism in precocious pre-pubertal testies that often presents with gross enlargement of testis. The study highlights the role of hypothyroidism in stimulating the increased proliferation of the sertoli cells that leads to clinical presentation of macroorchidism [8]. The manifestation of Ovulatory dysfunction in females accompanied by the formation of multiple cysts in cases of primary hypothyroidism may be due to several mechanisms including altered metabolism of oestrogen and its analogues, dysregulation of hypothalamic-pituitary-gonadal axis and an altered prolactin metabolism [7]. Precocious puberty is the term used for precocious progression of puberty in terms of secondary sexual characteristics. The established literature pronounces that consensus to investigate precocious puberty is different for boys and girls. For boys, the age is 9 years and that for a girl is 8 years. Checking the underlying etiology in such cases becomes a foremost task as it could dictate terms for prognosis and management [9]. The accurate underlying mechanism establishing grounds for precocious puberty in VWGS is unknown. Van Wyk and Grumbach proposed a hypothesis suggesting the involvement of uncertainty in feedback mechanism leading to dysregulated production of various hormones [10]. Recently using recombinant tools, it has been uncovered that human TSH can potentially interact with human FSH receptors to upregulate adenylyl cyclase. The study conducted on the ovaries of hamsters in China [11] suggests the association between extremely high concentrations of TSH seen in hypothyroidism and their action on FSH receptors that lead to precocity.

Long-standing hypothyroidism is believed to be involved in the pathology of VWGS. The most common cause regarded to cause hypothyroidism is autoimmune thyroiditis. In this condition, body's immune system attacks thyroid cells as if they were foreign bodies causing infection. This leads to dramatic elevation in TSH in the blood. Elevated TSH levels alongside the prepubertal levels of LH suggest VWGS. However, it is of utmost importance to understand and acknowledge the unique characters of our case. Opposed to our existing knowledge based on literature, our patient did not have bilateral multi-cystic ovaries. This divergence from the literature [12] not only provides us the acknowledgment of the syndrome's heterogeneity but also points at the possibility of diversity in VWGS's clinical presentation. It showcases that due to the possibility of diversity in its clinical presentation, a variety of varied symptoms and manifestations may present in investigations. Cases reported previously also showed similar results [13, 14].

## Conclusions:

Van Wyk-Grumbach Syndrome, a rare endocrine disorder affecting predominantly prepubertal boys and girls which presents with diffusely enlarged pituitary gland, multicystic ovaries in girls and macroorchidism in boys, precocious puberty and delayed bone age shows that presentation of every case clinical is unique with certain shared characteristics. Our case report highlights the frequent findings of this disorder that were reported in our tertiary healthcare hospital. As we evaluate different cases of VWGS, we ultimately uncover a landslide medley of symptoms and clinical presentations representing its medical heterogeneity. As every case of Van Wyk-Grumbach Syndrome offers novel insights in its clinical presentation and recommends different approach for diagnosis, interventions and management, it ultimately paves way to understand the diversity in challenges it offers to patients and the healthcare workers in terms so as to limit surgical intervention to cases that aren't sufficiently treated with thyroid hormone replacement to avoid surgical misadventures.

## Figures and Tables

**Table 1:** Blood Investigations

**Investigation**	**Result**	**Biological reference range**
Hemoglobin	10.6g/dl	12.0-16.0
Hemoglobin %	73.03	
WBC total count	6300/cmm	4000-12000
Neutrophils	38.50%	50-70
Lymphocytes	56.10%	20-50
Eosinophils	1.10%	01-Jun
Monocytes	4.30%	01-Oct
Basophils	0%	0-1
Absolute neutrophils count	2.43x10^3/uL	2.00-8.00
Absolute lymphocytes count	3.54x10^3/uL	0.8.-7.00
Absolute eosinophil count	0.07x10^3/uL	0.02-0.80
Absolute monocytes count	0.27x10^3/uL	0.12-1.20
Absolute basophils count	0x10^3/uL	0.00-0.10
RBC	3.47 mil/cmm	4.4-5.8
HCT	28.40%	38-48
MCV	81.8 fm	75-96
MCH	30.5 pg	25-32
MCHC	37.3 g/dl	30-35
RDW	14.60%	11.6-13.7
Platelet count	167000/cmm	150000-450000
MPV	9.4 fl	06-Dec
